# Elevated liver fibrosis index FIB-4 is not reliable for HCC risk stratification in predominantly non-Asian CHB patients

**DOI:** 10.1097/MD.0000000000004602

**Published:** 2016-09-23

**Authors:** Münevver Demir, Friederike Grünewald, Sonja Lang, Christoph Schramm, Andrea Bowe, Vera Mück, Fabian Kütting, Tobias Goeser, Hans-Michael Steffen

**Affiliations:** Clinic for Gastroenterology and Hepatology, University Hospital of Cologne, Cologne, Germany.

**Keywords:** chronic hepatitis B infection, FIB-4, HCC risk stratification, hepatocellular carcinoma

## Abstract

We aimed to validate the liver fibrosis index FIB-4 as a model for risk stratification of hepatocellular carcinoma development in predominantly non-Asian patients with chronic hepatitis B infection seen at a tertiary referral center in Germany.

We retrospectively analyzed 373 adult patients with chronic hepatitis B infection. Patient demographics, hepatitis B markers, antiviral treatment, laboratory parameters, results from liver imaging and histology were recorded. Patients were divided into 2 groups according to their FIB-4 levels and their hazard ratios for developing hepatocellular carcinoma were analyzed adjusted for age, sex, body mass index, alcohol consumption, and antiviral medication.

Median follow-up was 8.7 years (range 1–21.3 years), 93% of patients were of non-Asian origin, and 64% were male. Compared with patients with a low FIB-4 (<1.25) patients with FIB-4 ≥1.25 showed a hazard ratio for incidence of hepatocellular carcinoma of 3.03 (95% confidence interval (CI): 1.24–7.41) and an adjusted hazard ratio of 1.75 (95% CI: 0.64–4.74). Notably, 68% of patients with liver cirrhosis and 68% of those who developed HCC during observation had a low FIB-4 (<1.25).

We could not confirm that a FIB-4 value ≥1.25 is a reliable clinical indicator for incidence of hepatocellular carcinoma in predominantly non-Asian patients with chronic hepatitis B. Further studies in geographically and ethnically diverse populations are needed to prove its utility as a predictive tool.

## Abstract 2

Supplemental Digital Content is available in the text

## 1 Introduction

Chronic hepatitis B virus (HBV) infection is a major global health burden—about 5% of the adult population worldwide is chronically infected with HBV.^[1]^ These patients have a substantially increased risk of liver cirrhosis and hepatocellular carcinoma (HCC).^[2–4]^ HCC is the fifth most common cancer worldwide and the third leading cause of cancer mortality.^[5,6]^ A trend of rising rates of HCC has been reported from several countries in Europe and North America.^[7–9]^ International guidelines for HCC management recommend offering surveillance to patients with liver cirrhosis or advanced chronic liver disease to detect HCC at early, potentially curable stages.^[10–14]^

However, it remains challenging to identify chronic hepatitis B (CHB) patients at a high risk for HCC development despite the known risk factors such as liver cirrhosis,^[15,16]^ coinfection with hepatitis C virus (HCV),^[17,18]^ hepatitis D virus (HDV) or alcohol abuse.^[19]^ In order to improve identification of CHB patients at high risk for HCC development an effective and routinely applicable predictive tool is needed. Recently, Suh et al^[20]^ investigated the possible role of FIB-4 as a clinical indicator for predicting future HCC among Korean hepatitis B surface antigen (HBsAg) carriers and were able to show that an elevated FIB-4 value is highly predictive for HCC incidence in this population. The FIB-4 index was initially developed for the noninvasive prediction of significant liver fibrosis and liver cirrhosis in patients with HIV/HCV-coinfection^[21]^ and was examined for the detection of liver fibrosis and cirrhosis in other chronic liver diseases including CHB^[22–25]^ or nonalcoholic fatty liver disease (NAFLD).^[26]^

The aim of this study was to validate the FIB-4 as a model for HCC risk stratification in an ethnically diverse and predominantly non-Asian CHB population from a tertiary referral liver center in Germany.

## 2 Patients and methods

We retrospectively evaluated the medical records of 655 adult (age ≥18 years) patients with CHB infection who presented to the Clinic for Gastroenterology and Hepatology, University Hospital of Cologne, Germany, between January 1994 and June 2011. Written informed consent from the participants and a votum of the local ethics committee were not obtained. In accordance with German law, a votum of a local ethics committee is not required to conduct strictly retrospective studies (paragraph 15, sentence 1, Nordrhein Medical Association's professional code of conduct from November 14, 1998 as amended on November 19, 2011). There is also no need to get written informed consent from the participants if the considered data was collected and analyzed retrospectively (paragraph 6, sentence 1, Health Data Protection Act of Nordrhein-Westfalen). CHB was defined as positive HBsAg and/or HBV DNA levels >10 IU/mL for at least 6 months. Patients were included if they had attended at least 3 CHB-related clinic visits within 24 months during the study window. Patients with only 2 visits were included if they had a surveillance period for at least 12 months and their survival status with or without HCC until April 2015 was known. A total of 282 patients did not fulfil the inclusion criteria and were excluded: 219 patients due to only 1 or 2 visits, 13 patients due to HCC diagnosis already at first presentation, and 51 patients due to viral coinfection (HCV n = 22, HDV n = 16 or HIV n = 13, respectively). Thus, a total of 373 CHB patients were eligible and could be included. For all included patients data regarding survival status and HCC development were updated by the reference date of April 30, 2015. Twenty-three patients were lost to follow-up, that is, their survival status with or without the development of HCC as of April 2015 remained unknown. These patients were included and analyzed according to their status until the last presentation.

The primary endpoint was the development of HCC during observation according to the underlying FIB-4 levels. Thus, every patient was censored once HCC was diagnosed independently from the ensuing therapy, for example, liver transplantation.

Patient demographics and HBV markers (HBsAg, hepatitis B envelope antigen (HBeAg) and quantitative HBV DNA based on a sensitive PCR assay), type and duration of antiviral treatment, body weight and height, hepatic panel, platelet counts, prothrombin time, alpha fetoprotein (AFP), alcohol consumption in grams per day and results from liver imaging from each visit as well as liver histology (scored according to the Desmet score ^[27]^) were recorded. The diagnosis of cirrhosis and fatty liver (FL) was based on histology or imaging examinations, that is, abdominal ultrasound, dynamic computed tomography (CT), or magnetic resonance imaging (MRI). HCC diagnosis was made according to the valid international recommendations for the respective time points of diagnosis.^[28–31]^ The chosen methods during the study period from 1994 to 2015 were biopsy, abdominal ultrasound, contrast enhanced computed tomography (CECT), MRI, contrast enhanced ultrasound (CEUS), and AFP measurement depending on the valid recommendation at the time of HCC diagnosis. Biopsies of suspected nodules were carried out only when diagnosis remained uncertain.

Baseline characteristics of the study population were analyzed by using descriptive statistics. The index date for measurement of baseline characteristics was defined as the window from the first presentation to our clinic up to 3 months apart. The FIB-4 index (age × aspartate aminotransferase/(platelet counts × alanine aminotransferase^1/2^))^[21]^ was calculated for each patient referring to the baseline data. Patients were divided into 2 groups according to their FIB-4 values using a cutoff <1.25 for a low risk (reference group) as previously proposed by Suh et al^[20]^ and a cutoff of ≥1.25 for an elevated to high risk. The baseline characteristics of the study population according to the respective FIB-4 groups were also analyzed.

Cox proportional hazards models were used to determine the association of a FIB-4 ≥1.25 with HCC, adjusted for age, sex, BMI, amount of alcohol consumption, and antiviral medication at baseline or during observation. The hazard ratios (HR) were compared in different subpopulations: subjects without liver cirrhosis or FL on imaging, absence of arterial hypertension, and low levels of HBV DNA (<2000 IU/mL). Patients lost to follow-up were included according to their status at the last presentation.

Data were analyzed using SPSS software version 22 (SPSS, IBM Inc, Chicago, IL). Numeric variables were expressed as means ± standard deviations (SD) or medians (ranges) and compared with the Mann–Whitney *U* test. Categorical variables were expressed as numbers and percentages and compared with the *χ*^2^ test. Cumulative incidences of HCC during surveillance were calculated using the Kaplan–Meier method. For all calculations a *P* value <0.05 was considered significant.

## 3 Results

Baseline characteristics of the study population are shown in Table 1. Concerning origin (not nationality) we analyzed a predominantly non-Asian population (93%); the majority of patients were from Southern Europe or the Middle East including North Africa (48%). The mean age at baseline was 41 years (± 13.6 years) and 239 (64%) patients were male. Median follow-up was 105 months (range 12–255 months) and 23 patients (7%) were lost to follow-up.

**Table 1:**
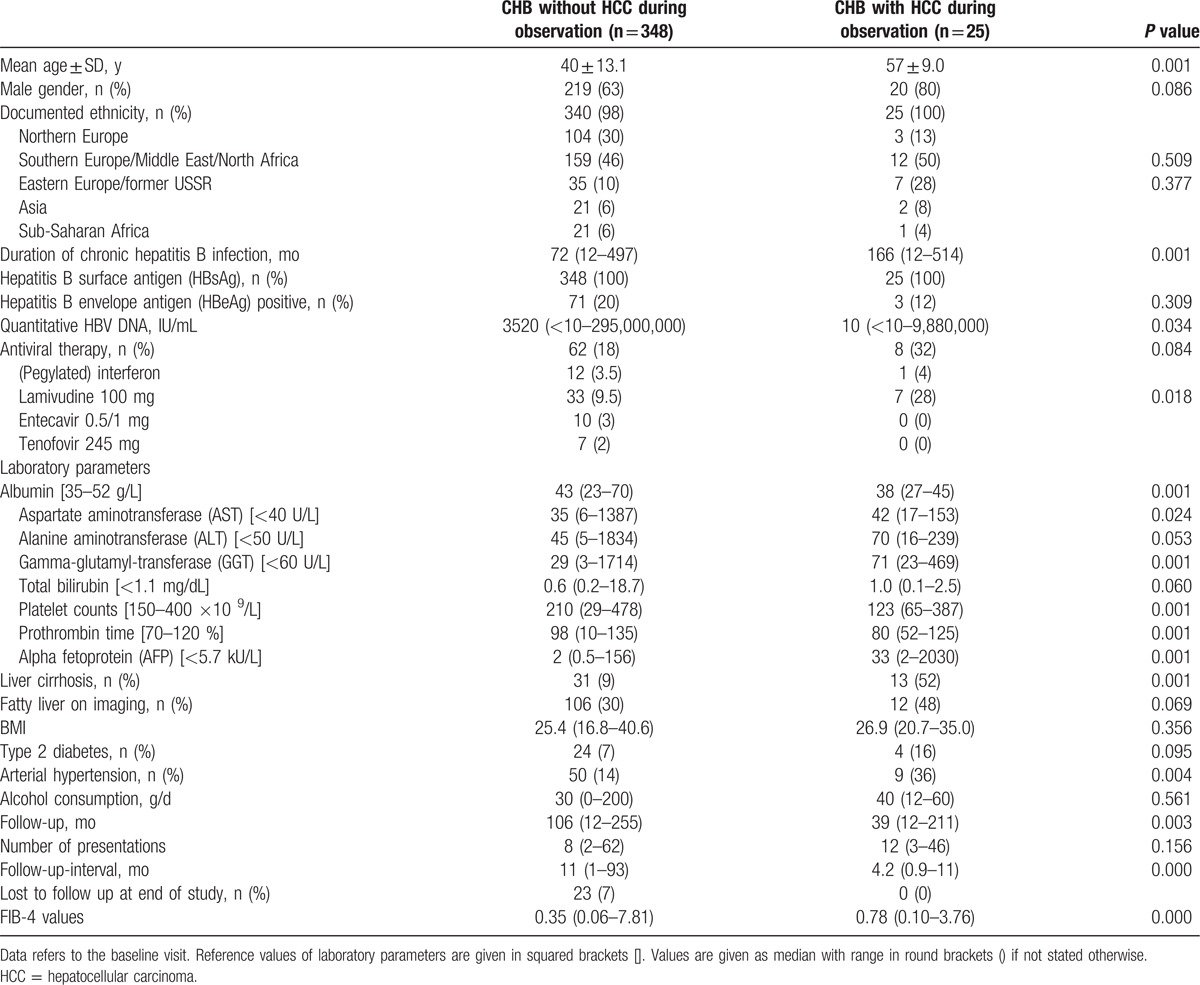
Baseline characteristics of the study population (n = 373).

Compared to CHB patients without HCC during observation, patients with HCC development under surveillance presented with statistically significant differences: a higher age (57 ± 9.0 years, *P* = 0.001), a longer duration of CHB infection (median 166 months, range 12–514 months, *P* = 0.001), more altered laboratory parameters (*P* = <0.025), and a higher rate of liver cirrhosis (52%, *P* = 0.001) as well as arterial hypertension (36%, *P* = 0.004) at baseline. A higher proportion was treated with lamivudine (38%, *P* = 0.018) and had lower levels of HBV DNA (10 IU/mL, range <10–9,880,000 IU/mL, *P* = 0.034). Furthermore, the median FIB-4 value was significantly higher in patients with HCC during observation compared with those without HCC; 0.78 (0.10–3.76) versus 0.35 (0.06–7.81; *P* = 0.000), respectively (Table 1).

Thirty patients (24 males) showed elevated FIB-4 levels (≥1.25). Of these 8 (27%) developed HCC during observation compared with 17 patients (5%) in the low risk group (<1.25). On the other hand, 68% of patients with liver cirrhosis and 17 patients (68%) who developed HCC during observation had a low FIB-4 level (<1.25). Since portal hypertension and splenomegaly may develop already in cases of advanced fibrosis the low median platelet count [70 ×10^9^/L] among patients with a FIB-4 ≥1.25 is not surprising despite a prevalence of only 46% confirmed cases of definite cirrhosis (Table 2).

**Table 2:**
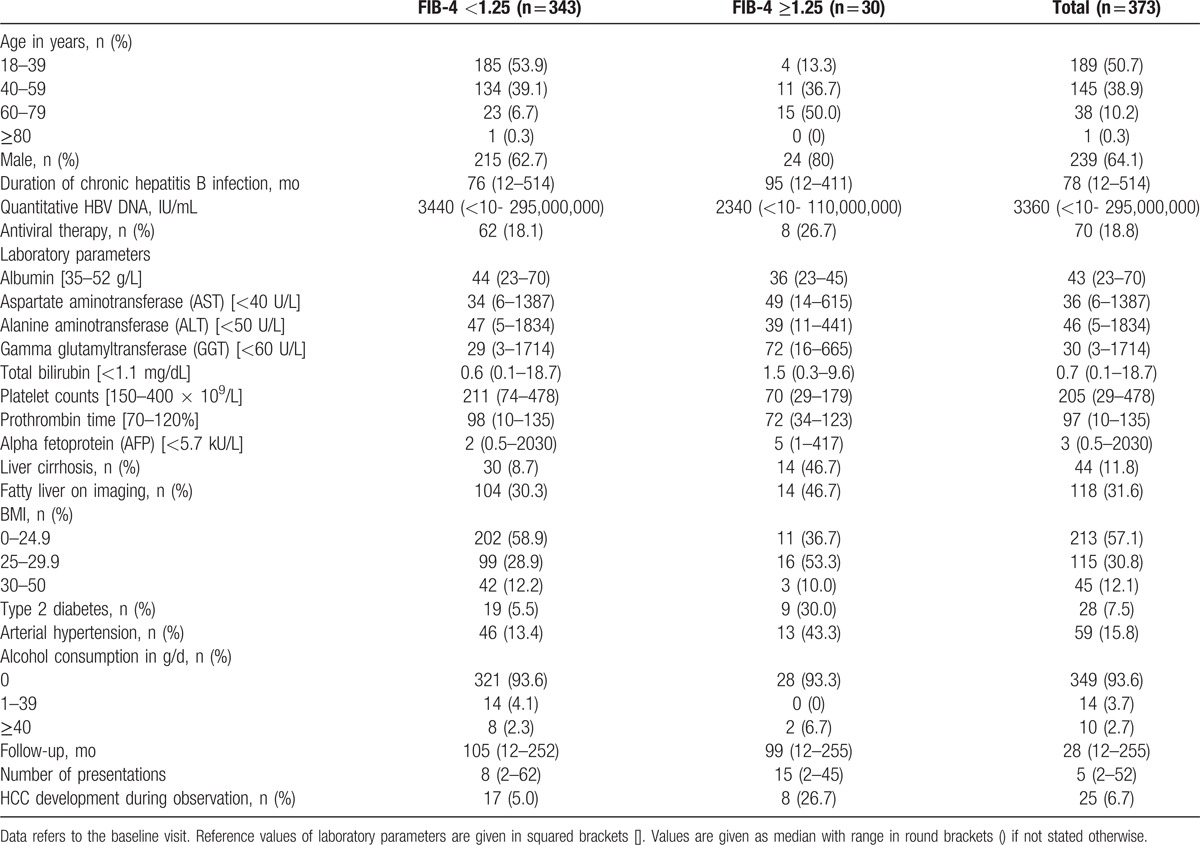
Baseline characteristics according to FIB-4 groups.

**Figure 1:**
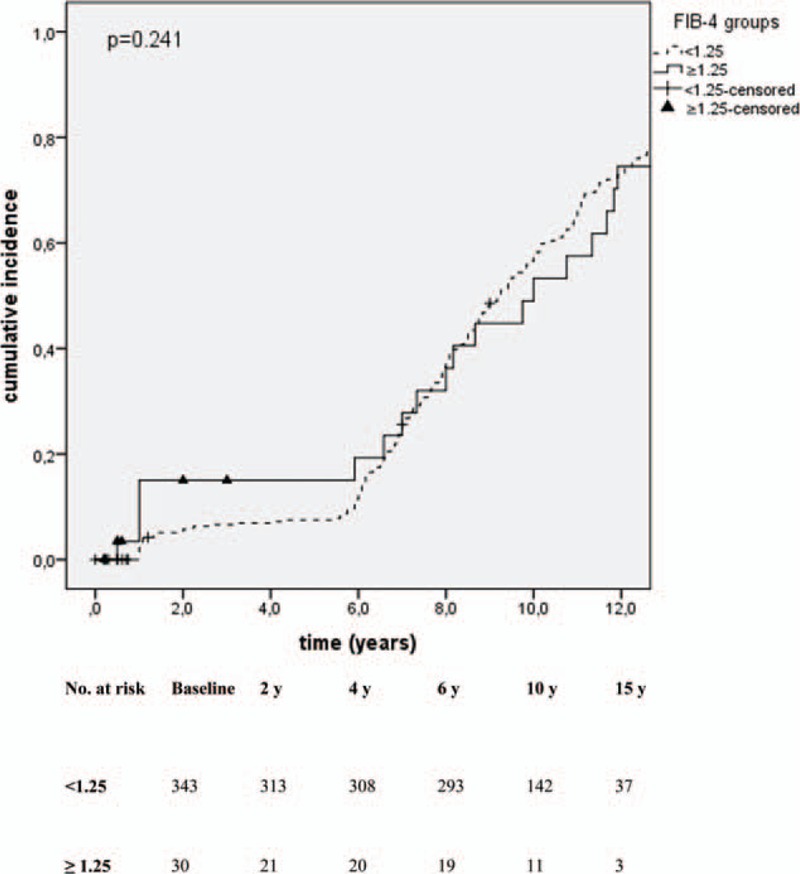
Kaplan–Meier curve for HCC incidence by FIB-4 groups (n = 373). HCC diagnoses during the observation period are marked with crosses (FIB-4 <1.25, broken line) and with closed triangles (FIB-4 ≥1.25, bold line). HCC = hepatocellular carcinoma.

Compared with the low risk group (FIB-4 <1.25; reference group) patients with FIB-4 ≥1.25 showed a HR of 3.03 (95% CI: 1.24–7.41) for HCC incidence. After adjustment for age, sex, BMI, amount of alcohol consumption, and antiviral medication for HBV patients with FIB-4 ≥1.25 showed an adjusted HR (aHR) of 1.75 (95% CI: 0.64–4.74) for HCC incidence compared with the reference group. None of these results were statistically significant (Table 3, Fig. 1).

**Table 3:**
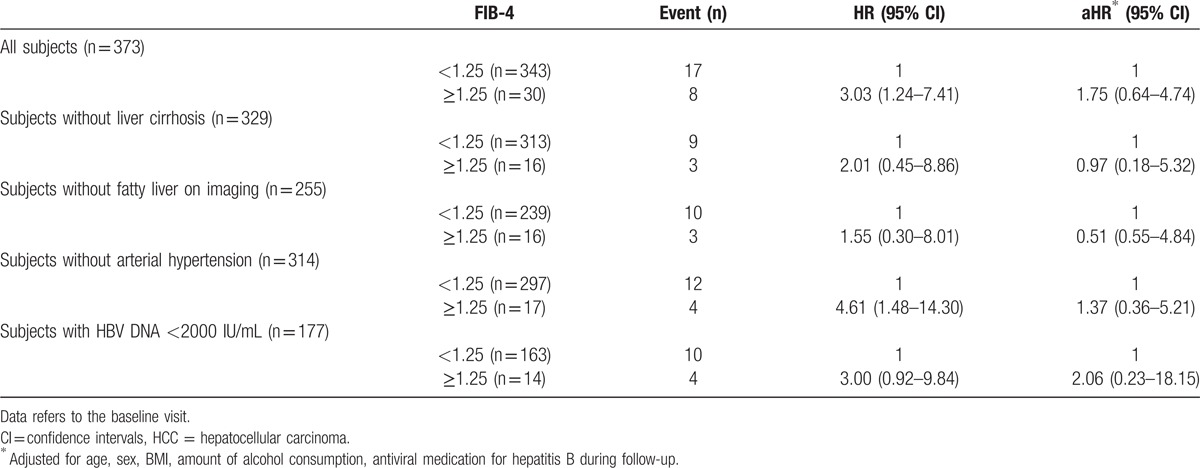
Cox proportional hazards models for HCC incidence.

Referring to the baseline data, neither the subpopulation without liver cirrhosis or FL on imaging nor the subjects without arterial hypertension showed significant aHR for developing HCC according to their FIB-4 levels (Table 3).

We additionally validated the FIB-4 index for the exclusion of severe fibrosis using the previously published cutoffs by Sterling et al.^[21]^ The analysis was performed in a subset of 252 patients for whom liver biopsies were available and who were not coinfected with HCV, HDV, or HIV or had HCC at initial presentation. FIB-4 was assessed at the time of liver biopsy or within 90 days around liver biopsy. The exclusion of significant fibrosis or liver cirrhosis at a cutoff <1.45 resulted in a specificity of 88% and a negative predictive value (NPV) of 81%, and the prediction of significant fibrosis or liver cirrhosis at a cutoff >3.25 showed a sensitivity of 26% and a positive predictive value (PPV) of 82%. The diagnostic accuracy was 68% and the area under the curve (AUC) 0.73 (95% CI: 0.65–0.80). Fifty-three percent of patients with confirmed F3/F4 on liver biopsy were misclassified as having F0-F2 according to FIB-4 (Supplementary Table 1).

## 4 Discussion

The aim of this study was to validate the FIB-4 as a model for HCC risk stratification in CHB patients as recently proposed by Suh et al.^[20]^ Therefore, we retrospectively analyzed a cohort of 373 predominantly non-Asian CHB patients. The outcome of interest was the relationship between the development of HCC during the study period and the individual underlying FIB-4 levels.

We found that the median FIB-4 value was significantly higher in patients with HCC development during observation compared with those without; 0.78 (0.10–3.76) versus 0.35 (0.06–7.81; *P* = 0.000), respectively. Furthermore, 27% of patients with an elevated FIB-4 (≥1.25) developed HCC during observation compared with 5% in the low risk group (<1.25). Both observations indicate that a FIB-4 ≥1.25 may be predictive for HCC incidence in CHB patients. However, we also found that, compared with patients with a low FIB-4 (<1.25), patients with FIB-4 ≥1.25 showed a HR for HCC incidence of 3.03 (95% CI: 1.24–7.41) and an aHR of 1.75 (95% CI: 0.64–4.74), respectively. None of these were statistically significant; thus, we could not confirm a predictive role of an elevated FIB-4 (≥1.25) for future HCC development in our study population.

Our results stand in clear contrast to the recently reported promising findings by Suh et al, who identified a subpopulation of subjects with high FIB-4, who are at high risk for future HCC incidence. In this retrospective cohort study of 986 Korean HBsAg carriers subjects with FIB-4 1.7 to 2.39 and ≥2.4 showed aHR for HCC incidence of 4.57 (95% CI: 1.50–13.92) and 21.34 (95% CI: 7.73–58.92), compared to those with FIB-4 <1.25.^[20]^

The FIB-4 index was initially developed in HIV/HCV coinfected patients to exclude significant fibrosis at a cutoff <1.45 (sensitivity 70%, NPV 90%) and predict severe fibrosis with a cutoff >3.25 (specificity 97%, PPV 65%).^[21]^ Given that a high FIB-4 reflects underlying liver cirrhosis, which is the main risk factor for HCC development,^[29]^ it is not surprising that the FIB-4 failed to predict HCC incidence in our study cohort. Despite the fact that the chosen cutoff for an elevated/high FIB-4 was lower than the proposed cutoff for the noninvasive detection of advanced liver fibrosis or cirrhosis (≥1.25 vs >3.25), 68% of patients with liver cirrhosis (being at high risk) were assigned to the low FIB-4 group (<1.25). Moreover, 68% of patients who developed HCC during observation allegedly had a low risk had they been classified solely by their FIB-4 levels (<1.25). Translating our findings into clinical practice would imply that two-thirds of our high-risk patients would have been misclassified as having a low risk without the need for regular surveillance, with potentially deleterious consequences for the individual, had the FIB-4 been applied without discretion.

Our results raise the question whether the FIB-4 is sufficiently accurate to predict advanced fibrosis or cirrhosis in predominantly non-Asian CHB populations. Mallet et al investigated the FIB-4 in comparison to liver biopsy in a French population of 138 CHB patients and discriminated none or moderate (F0-F2) from severe fibrosis (F3-F4) at a cutoff ≤1.45. They found a sensitivity of 71%, a specificity of 73%, a NPV of 86%, and a PPV of 52%. However, 35% (11/41) of patients with F3/F4 on liver biopsy were misclassified as none or moderate fibrosis (F0-F2) according to FIB-4.^[32]^ In a European multicenter study including 253 patients with CHB, Sebastiani et al^[33]^ reported that the FIB-4 index excluded significant fibrosis at a cutoff <1.45 with a sensitivity of 70%, a NPV of 60%, and a diagnostic accuracy of only 67%. Against the backdrop of this evidence we additionally validated the FIB-4 index for the exclusion of severe fibrosis using the previously published cutoffs by Sterling et al.^[21]^ The diagnostic accuracy was only 68% with an area under the curve (AUC) of 0.73 (95% CI: 0.65–0.80). The most important finding was that 53% of patients with confirmed F3/F4 on liver biopsy were misclassified as having F0-F2 according to FIB-4 (Supplementary Table 1). These results are similar to those from other European centers,^[32,33]^ supporting the assumption that the FIB-4 is not accurate enough to reliably exclude advanced fibrosis or cirrhosis in mainly non-Asian CHB populations.^[32,33]^ As patients with advanced fibrosis or cirrhosis are at highest risk for HCC development,^[29]^ the reliable identification of them by FIB-4 is a prerequisite for being clinically useful as a HCC risk stratification tool.

According to the baseline characteristics of our study population, there are some statistically significant differences between patients who developed HCC during observation compared to those who did not (Table 1). Patients with HCC incidence were older (57 ± 9.0 years, *P* = 0.001), had a longer duration of CHB infection (median 166 months, range 12–514 months, *P* = 0.001), presented higher levels of ALT and AST (median 70 U/L, range 16–239 U/L, *P* = <0.05 and 42 U/L, range 17–153 U/L, *P* = <0.024, respectively), reflecting a higher inflammatory activity,^[34,35]^ as well as higher levels of AFP (median 33 kU/L, range 2–2030 kU/L, *P* = 0.001) and a higher rate of liver cirrhosis (52%, *P* = 0.001) and arterial hypertension (36%, *P* = 0.004) at baseline. Interestingly, the differing factors are mostly well-known risk factors for HCC development. An age above 50 years is considered one of the most important risk factors for the development of HCC in patients with advanced liver disease.^[15]^ A long duration of CHB infection and a high inflammatory activity are risk factors for HCC, by increasing the risk for liver cirrhosis,^[36]^ which itself is the major risk factor for HCC incidence.^[29]^ In approximately 70% of patients with HCC, AFP is expressed in tumor tissue and can be found in the serum.^[37]^ With an increase of AFP ≥400 ng/mL, the existence of an HCC can be expected in 95% of cases.^[38]^ However, it has to be noted that at lower range AFP has a limited sensitivity for HCC diagnosis.^[39]^ Another statistically significant difference was that patients with HCC incidence had a lower HBV DNA at baseline (10 IU/mL, range <10–9,880,000 IU/mL; *P* = 0.034). As discussed in a previous paper on CHB management there is a controversy whether a low HBV DNA reduces the risk for HCC.^[40]^ Recent studies have shown that a reduction of HBV DNA to low or undetectable levels reduces the risk of liver-related events and HCC.^[41–43]^ However, there is also rising evidence that adequate long-term viral suppression does not reduce the incidence of HCC. In a multicenter study of 744 CHB patients in a Western population by Arends et al, 14 patients developed HCC, of whom 12 had achieved virological response (HBV DNA <80 IU/mL) before HCC development.^[44]^ Similar results were reported by Papatheodoridis et al,^[45]^ who analyzed 818 HBeAg negative CHB patients from Greece and found that virological on-therapy remission did not significantly affect the incidence of HCC. Considering this evidence it seems reasonable why patients who developed HCC during observation were concurrently those who had a statistically significant lower HBV DNA. Another potential explanation is that some of the patients who developed HCC had inactive hepatitis B, at least at baseline.

The observed baseline characteristics also imply that the analyzed patient cohort was broadly representative for a CHB population of a European liver center. Hence, the failure of FIB-4 as a predictive tool for HCC incidence cannot be attributed to an investigation of a potentially atypical study population.

Our study population shows some differences when compared with the population of Suh et al.^[20]^ Our patients were younger (41 years vs 53 years) and mostly male (64% vs 55%). Nineteen percent of our patients received antiviral treatment at baseline compared to 4% in the study by Suh et al. The higher rate of antiviral treatment in our study could have led to lower AST and ALT levels with the consequence of lower FIB-4 values in our population. This could at least partially explain the small proportion of patients with FIB ≥1.25 in our study. Although total mean AST and ALT values in the study by Suh et al were similar to our findings (35 U/L vs 36 U/L and 40 vs 46 U/L, respectively), 10.5% of the study population had considerably elevated transaminases with a mean AST of 92 ± 146 U/L and a mean ALT of 107 ± 194 U/L which consecutively resulted in a high FIB-4 (≥2.4; high-risk group). This observation might also raise the question whether the proposed cutoffs by Suh et al^[20]^ are too high for patients under antiviral therapy as AST and ALT usually normalize during treatment leading to low or lower FIB-4 values. Another difference is that the proportion of patients with liver cirrhosis or fatty liver was higher in our study population than in the population of Suh et al (11.8% vs 9.9% and 31.6% vs 24.3%, respectively). This may have influenced our results. However, even if the proportion of patients with liver cirrhosis or fatty liver in our predominantly non-Asian study population differs from the Asian population in the study by Suh et al, the missing predictive value of a FIB-4 ≥1.25 remains unaffected either related to ethnicity or differences in lifestyle in different geographical regions.

Our study has several limitations. Due to its retrospective design we were not able to control time intervals of follow-up visits. In order to ascertain a sufficient follow-up period in our study, we excluded all patients who had only one or two short timed visits at our clinic, which resulted in an exclusion of 218 patients. It might also have led to a higher disease burden in our study population. This may be reflected by the fact that we had a relatively high HCC incidence of 6.7% in our study population. In CHB patients, annual HCC incidences are 4% to 6% in Asian populations and less than 1% in Caucasian populations.^[46]^ Most of the HCCs in our study were observed within the first 3 to 4 years of the observation period. Since these patients suffered significantly longer from CHB infection already at study entry this probably relates to a lead-time bias. Another limitation is the generalizability of data derived from a tertiary liver center to the community.

The strength of this study is that patient data were extracted from medical records and analyzed by experienced physicians and were not derived from medical service claims, suggesting a high clinical accuracy of the analyzed data. For all measures of interest, data were complete including the availability of serial HBV DNA, HCV RNA, HIV RNA, and HDV RNA based on sensitive PCR assays. To ascertain a reliable and adjusted database for analysis, we strictly excluded confounding factors such as viral coinfection and HCC at first presentation as well as patients with short timed visits although this led to an exclusion of 43% of our initially identified CHB cohort. We had a fairly long duration of follow-up (median 8.7 years; range 1–21.3 years) with a low lost to follow-up rate of 7%.

To the best of our knowledge, we are the first to validate the FIB-4 index for HCC risk stratification in a predominantly non-Asian CHB population. In conclusion, we could not prove a predictive role of a high FIB-4 for HCC incidence in an ethnically diverse non-Asian CHB population. Further studies in geographically and ethnically different populations are needed to clarify the potential role for FIB-4 as a noninvasive tool for the prediction of HCC incidence in CHB patients.

## Supplementary Material

Supplemental Digital Content
