# Supplementary material for: Elevated liver fibrosis index FIB-4 is not reliable for HCC risk stratification in predominantly non-Asian CHB patients
Source: Medicine (Baltimore). 2016 Sep 23;95(38):e4602. doi: 10.1097/MD.0000000000004602 (PMC5044885; doi:10.1097/MD.0000000000004602)
Supplement: Supplemental Digital Content [file medi-95-e4602-s001.docx]

Supplementary Table 1: Diagnostic performance of the original FIB-4 Index for the noninvasive assessment of liver fibrosis (n=252)

|  | FIB-4  <1.45 | FIB-4  1.45-3.25 | FIB-4  >3.25 | All patients |
| --- | --- | --- | --- | --- |
| All patients, n (%) | 190 (75.4) | 40 (15.9) | 22 (8.7) | 252 |
| F0-F2, n (%) | 154 (83.7) | 26 (14.1) | 4 (2.2) | 184 |
| F3-F4, n (%) | 36 (52.9) | 14 (20.6) | 18 (26.5) | 68 |
| Sensitivity, % | 47.1 |  | 26.5 |  |
| Specificity, % | 88.4 |  | 97.8 |  |
| PPV, % | 51.6 |  | 81.8 |  |
| NPV, % | 81.5 |  | 87.3 |  |
| Diagnostic accuracy | 0.68 | | | |
| AUC (95% CI) | 0.73 (0.651-0.80) | | | |
